# Magnetite Mineralization inside Cross-Linked Protein Crystals

**DOI:** 10.1021/acs.cgd.2c01436

**Published:** 2023-04-28

**Authors:** Mariia Savchenko, Victor Sebastian, Modesto Torcuato Lopez-Lopez, Alejandro Rodriguez-Navarro, Luis Alvarez De Cienfuegos, Concepcion Jimenez-Lopez, José Antonio Gavira

**Affiliations:** † Departamento de Química Orgánica, Facultad de Ciencias, Unidad de Excelencia de Química Aplicada a Biomedicina y Medioambiente (UEQ), Universidad de Granada, 18002 Granada, Spain; ‡ Laboratorio de Estudios Cristalográficos, Instituto Andaluz de Ciencias de la Tierra (Consejo Superior de Investigaciones Científicas-Universidad de Granada), Avenida de las Palmeras 4, 18100 Armilla, Granada, Spain; § Department of Chemical Engineering and Environmental Technology, Instituto de Nanociencia y Materiales de Aragón (INMA), CSIC-Universidad de Zaragoza, Zaragoza 50009, Spain; ∥ Networking Research Center on Bioengineering Biomaterials and Nanomedicine (CIBER- BBN), Madrid 28029, Spain; ⊥ Departamento de Física Aplicada, Facultad de Ciencias, Universidad de Granada, 18002 Granada, Spain; # Instituto de Investigación Biosanitaria ibs, Granada 18012, Spain; ∇ Departamento de Mineralogía y Petrología, Facultad de Ciencias, Universidad de Granada, 18002 Granada, Spain; ○ Departamento de Microbiología, Facultad de Ciencias, Universidad de Granada, Campus de Fuentenueva s/n, 18002 Granada, Spain

## Abstract

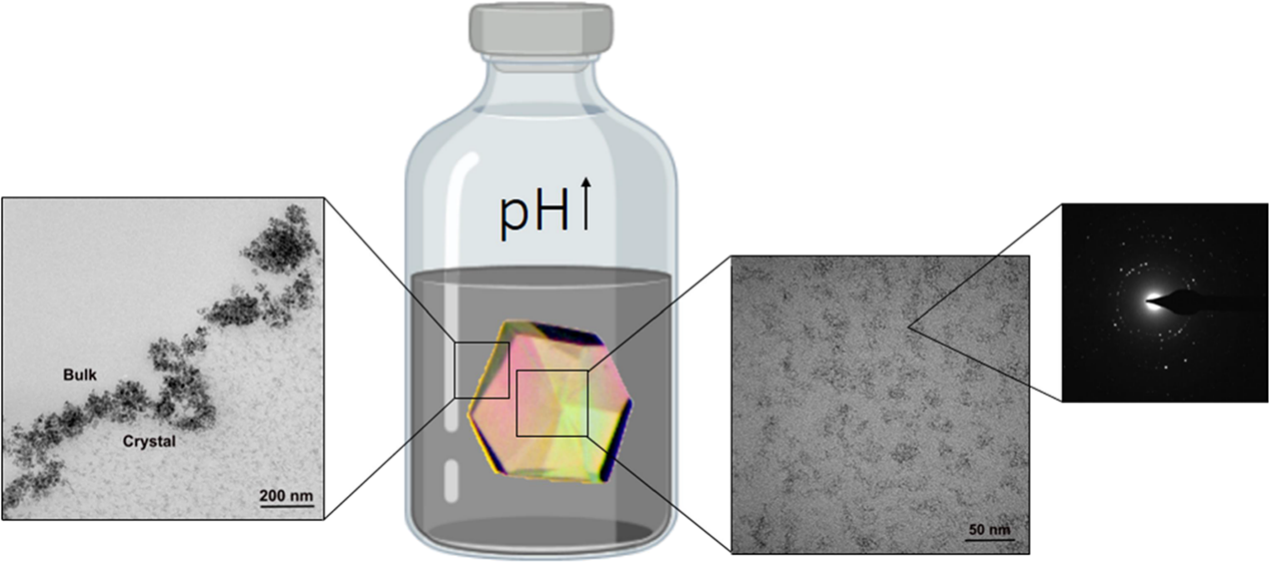

Crystallization in confined spaces is a widespread process in nature that also has important implications for the stability and durability of many man-made materials. It has been reported that confinement can alter essential crystallization events, such as nucleation and growth and, thus, have an impact on crystal size, polymorphism, morphology, and stability. Therefore, the study of nucleation in confined spaces can help us understand similar events that occur in nature, such as biomineralization, design new methods to control crystallization, and expand our knowledge in the field of crystallography. Although the fundamental interest is clear, basic models at the laboratory scale are scarce mainly due to the difficulty in obtaining well-defined confined spaces allowing a simultaneous study of the mineralization process outside and inside the cavities. Herein, we have studied magnetite precipitation in the channels of cross-linked protein crystals (CLPCs) with different channel pore sizes, as a model of crystallization in confined spaces. Our results show that nucleation of an Fe-rich phase occurs inside the protein channels in all cases, but, by a combination of chemical and physical effects, the channel diameter of CLPCs exerted a precise control on the size and stability of those Fe-rich nanoparticles. The small diameters of protein channels restrain the growth of metastable intermediates to around 2 nm and stabilize them over time. At larger pore diameters, recrystallization of the Fe-rich precursors into more stable phases was observed. This study highlights the impact that crystallization in confined spaces can have on the physicochemical properties of the resulting crystals and shows that CLPCs can be interesting substrates to study this process.

## Abstract 2

Cross-linked protein crystals provide a well-ordered decorated channel array to study inorganic precipitation in confined space of modulable size and physicochemical properties.

## 1 Introduction

There are a number of very relevant processes (nucleation, growth, phase transformation, etc.) that occur in confined spaces, such as those taking place during biomineralization,^1,2^ methods to form nanomaterials,^3,4^ frost heave scale formation,^5^ etc., that require our attention and dipper understanding of how crystallization processes in these small cavities occur. These processes can provide valuable information about the weathering and decay of construction materials,^6^ contribute to developing strategies for the remediation of contaminants,^7^ and have a significant impact on different research areas such as pharmaceuticals, material science, nanomaterials, biomineralization, and geochemistry.^5,8^ Additionally, crystallization in confinement can alter nucleation rates, as well as crystal size, polymorph, morphology, and orientation,^5,9−13^ so research in this area contributes to increasing knowledge in the field of crystallization, and therefore, it is important from a fundamental point of view. One example of a nanocrystal formed in a confined space is the magnetosome of magnetotactic bacteria. Although the biomineralization process of the magnetosome is not well known, it has been proven that the nucleation and growth of the magnetic crystal forming the magnetosome occur in confined vesicles, modulated by the interaction with different magnetosome membrane proteins, resulting in crystals with a homogeneous size and morphology with excellent magnetic properties.^14^ In particular, the formation of a ferrihydrite metastable precursor has been demonstrated during this biomineralization process, which later crystallizes into the stable mineral form (i.e., magnetite).^15−17^ Little is, however, known about the formation of these precursors, their stability, their maturation, and whether or not their formation is linked to the confinement. A better understanding of the role of confinement in magnetite formation is not only interesting from a fundamental point of view, but also to produce novel magnetite nanoparticles with defined sizes that might be of potential interest in technological application.

One of the challenges in the study of crystallization in confined spaces is the selection of an appropriate experimental setup and analysis techniques. In fact, understanding the effects of confinement relies on the ability to analyze those effects on crystals isolated from this environment or preferably, to perform the analysis in situ, directly in the confined space. This analysis, in most of the cases, is not simple to do and is restrained to the use of few techniques such as electron microscopies, X-ray tomography, and X-ray or neutron diffraction.^5^ Furthermore, to clearly understand the effects of confinement on crystallization, simultaneous analysis of the solution surrounding the confined space must be performed.

Another challenge is the nature or type of the confined space, which has to be able to modify the kinetics or thermodynamics of the crystallization by reducing the three dimensions of the system, preferably in a well-defined manner. Different systems have been studied, such as droplets/microfluidic devices,^18−23^ as well as materials that have pores of different sizes,^24^ like polymeric matrices,^25−28^ microporous and mesoporous materials, glasses,^29,30^ or carbon nanotubes.^31−33^ In this context, porous protein crystals have the potential to become an interesting confined space to study crystallization thanks to their well-defined and regular distribution of pores (solvent channels), as well as their defined chemical composition reproduced regularly in the crystal lattice.^34,35^

Although protein crystals are mechanically unstable, cross-linking these crystals can improve their stability and maintain their shape and catalytic activity or being used as heterogeneous catalysts even after being dry for preservation.^36−38^ In fact, cross-linked protein crystals (CLPCs) have been explored for the formation of new inorganic or hybrid materials^39^ by a process that implies metal or chemical coordination with the amino acids exposed to the solvent channels and a subsequent reduction or precipitation of the particles.^34^ This technique has been used to obtain well-ordered arrays of metallic nanoparticles of Pd/Pt,^40^ Ag,^41−43^ Au,^41,43−45^ Co/Pt,^46^ and CdS quantum dots,^47^ giving rise to composite protein crystals with new catalytic and optical properties.

**Figure 1:**
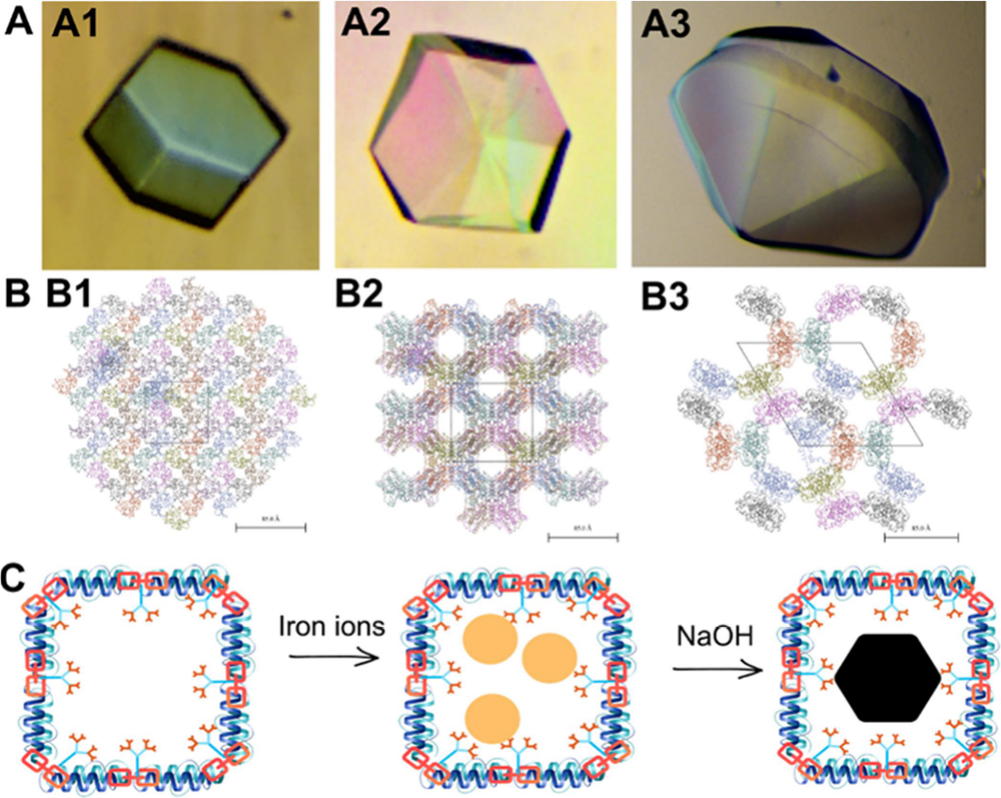
(A) Optical micrograph of each protein crystal: A1—lysozyme; A2—glucose isomerase; A3—lipase. (B) Crystal structures: B1—lysozyme; B2—glucose isomerase; B3—lipase. (C) Schematic picture describing magnetite precipitation inside pores of CLPCs.

Herein, as a model to understand better crystal formation in confined spaces, we have studied the nucleation and growth of magnetite nanoparticles inside the channels of CLPCs formed by coprecipitation of ferrous and ferric iron in alkaline aqueous solution. CLPCs act as reaction vessels in which the nucleation and growth of magnetite can be affected by a confined growth modulated by chemical interactions. CLPCs of different pore sizes such as tetragonal lysozyme (2.0 nm Ø), orthorhombic glucose isomerase (3.5 nm Ø), and hexagonal lipase (8.0 nm Ø) have been used in this study (Figure 1). The characterization of magnetite particles has been carried out in situ directly from nanometric slides of CLPCs visualized by transmission electron microscopy (TEM). High-resolution transmission electron microscopy (HR-TEM) of thin slides of each mineralized CLPC was carried out to characterize the nanoparticles and for detection of diffraction using selected area electron diffraction (SAED).

## 2 Materials and Methods

### 2.1 Reagents and Materials

#### 2.1.1 Reagents for Protein Crystallization and Cross-Linking

Lysozyme (62971, HEWL, three-times crystallized powder), sodium acetate (AcONa 99%), sodium chloride (NaCl 99%), magnesium chloride (MgCl_2_ ≥98%), monopotassium phosphate (KH_2_PO_4_ ≥99.0%), monosodium phosphate (NaH_2_PO ≥99.0%), HEPES (≥99.5%), NaH_2_PO TRIS (99.9%), and glutaraldehyde solution (Grade II, 25% in H_2_O) were purchased from Sigma-Aldrich (Madrid, Spain). Glucose isomerase D-xylose-ketol-isomerase from *Streptomyces rubiginosus* was purchased as a crystal suspension from Hampton Research (HR7–100). Lipase (*Aspergillus sp*) was purchased from Biocon as Biolipasa-L (Barcelona, Spain).

Lysozyme was dissolved in 50 mM AcONa, dialyzed (24 h) against 50 mM AcONa (pH 4.5) in a ratio 1:1000 at 4 °C and concentrated by centrifugation at 4 °C (*g* = *5000/25 min) to ≈150 mg mL^–1^, using a theoretical value for the extinction coefficient at 280 nm of 2.56 mL mg^–1^. Glucose isomerase was dialyzed for 24 h against HEPES 100 mM pH 7.0 at a ratio 1:1000 at 4 °C and concentrated by centrifugation at 4 °C (*g* = *5000/1 h) to ≈75 mg mL^–1^ using a theoretical value for the extinction coefficient at 280 nm of 1.074 mL mg^–1^. Lipase was dialyzed for 24 h against Milli-Q water at a ratio 1:1000 at 4 °C and also concentrated by centrifugation at 4 °C (*g* = *5000/4 h) to ≈40 mg mL^–1^ using a theoretical value for the extinction coefficient at 280 nm of 1.2 mL mg^–1^. Prior to the experimental setup, all the protein solutions were filtered through a 0.45 μm pore-size filter membrane system (Millipore).

The precipitant solutions (NaCl, MgCl_2_, K/NaH_2_PO_4_) with desirable concentration were obtained from their stock solution by diluting with appropriate buffer (50 mM AcONa pH 4.5; 0.01 M HEPES pH 7.0; 0.1 M TRIS pH 7.0). Then, the solutions were filtered through a 0.45 μm pore-size filter membrane system (Millipore).

Agarose D-5 was purchased from Hispanagar (Madrid, Spain). Sol of agarose with desirable concentration was obtained by dissolving agarose in Milli-Q water and heating at 90 °C until obtaining a homogeneous transparent solution. Then the solution was cooled down to 70 °C.

#### 2.1.2 Reagents for Magnetite Precipitation

NaHCO_3_, Na_2_CO_3_, NaOH, Fe(ClO_4_)_2_, and FeCl_3_ were purchased from Sigma-Aldrich. The stock solutions, NaHCO_3_/Na_2_CO_3_ (0.15 M/0.15 M), NaOH (1 M), Fe(ClO_4_)_2_ (0.5 M), and FeCl_3_ (1 M), were prepared with deoxygenated water inside an anaerobic chamber (Coy Laboratory Products, Grass Lake, MI) filled with 4% H_2_ in N_2_.

### 2.2 Production of CLPCs

Protein crystals were obtained by a batch method. Lysozyme crystals were grown in 0.2% w/v agarose using 30 mg mL^–1^ lysozyme solution, 3% NaCl, and 50 mM NaOAc buffer pH 4.5. Glucose isomerase crystals were obtained in 0.1% w/v agarose using 30 mg mL^–1^ of protein solution, 0.2 M solution of MgCl_2_, and 0.01 M HEPES buffer pH 7.0. Lipase crystals were obtained in 0.2% w/v agarose using 15 mg mL^–1^ lipase solution and 0.3 M solution of K/NaH_2_PO_4_ and 0.1 M TRIS buffer pH 7.0. All concentrations are final after mixing all components.

CLPCs (cross-linked protein crystals): CLLCs (cross-linked lysozyme crystals), CLGICs (cross-linked glucose isomerase crystals) and CLLPCs (cross-linked lipase crystals) were obtained by diffusion of isotonic 5% v/v glutaraldehyde solution through the agarose gel of the batches at 20 °C for 24 h. For enhanced cross-linking, in case of glucose isomerase and lipase crystals, they were additionally soaked in 10% v/v glutaraldehyde solution for 24 h at 20 °C. To avoid an osmotic shock, before using the CLPCs, crystals were sequentially transferred through a series of precipitant solutions at lower precipitant concentration than used for crystallization.

### 2.3 In Situ Formation of Magnetite

#### 2.3.1 Precipitation of Magnetite in CLPCs

Two types of experiments (Types 1 and 2) were done to study magnetite precipitation in different environments. In both cases, CLPCs (lysozyme, glucose isomerase, and lipase crystals) were placed in a glass vial inside an anaerobic COY chamber filled with 4% H_2_ in N_2_.

The master solution was prepared inside the anaerobic chamber in 10 mL to a final concentration of 2.78 mM Fe(ClO_4_)_2_, 3.5 mM NaHCO_3_, 3.5 mM Na_2_CO_3_, and 5.56 mM FeCl_3_. The procedure is described by Perez-Gonzalez et al.^48^

**Figure 2:**
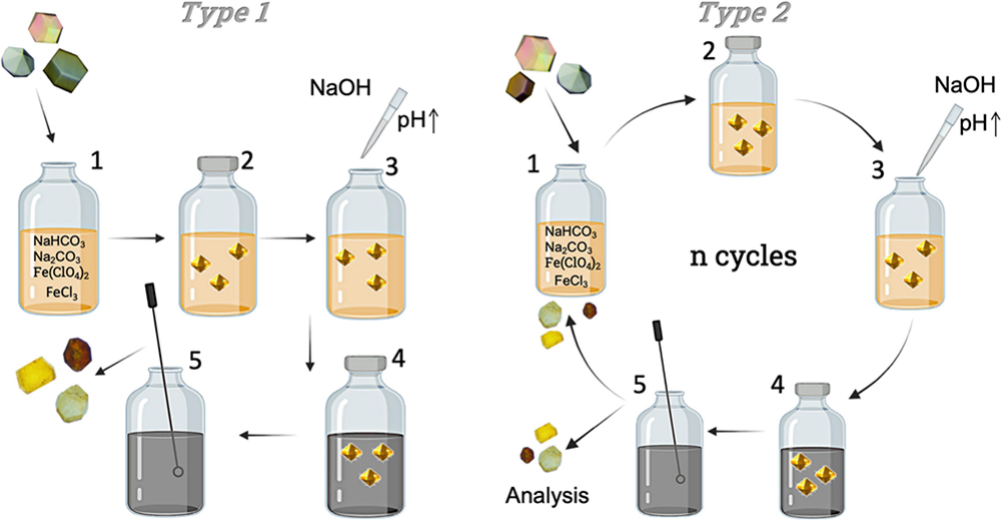
Event sequence in protocol *Type 1*: (1) CLPCs are added to the master solution (NaHCO_3_/Na_2_CO_3_, Fe(ClO_4_)_2_, and FeCl_3_), (2) incubation of the CLPCs to allow iron diffusion, (3) initiation of the precipitation by the addition of NaOH and changing the pH to 12.5, (4) precipitation of magnetite, and (5) fishing out the CLPCs for the analysis. *Type 2* “Cycles”: (1) 100 CLPCs are added in the master solution, (2) incubation, (3) initiation, (4) precipitation of magnetite, and (5) at least two CLPCs are preserved for characterization and the rest of the crystals are placed in a fresh master solution to start a new cycle, up to nine cycles.

Figure 2 represents schematically the two types of experiments. The initial one, named Type 1, in which CLPCs were exposed to the magnetite precipitation procedure only one time and Type 2 for which iron solution was freshly renewed “*n*” times.

##### 2.3.1.1 Type 1

CLPCs (min three crystals of each protein) were placed in a vial with the master solution and incubated for 4 to 12 days to allow iron ions to diffuse within crystal pores. Each experiment was set up in triplicate.

Precipitation was triggered by increasing the pH adding NaOH under anaerobic conditions (COY chamber), and the pH was recorded after each experiment. In the initial phase of the experiment, the pH was varied from 8.0 to 12.5 by increasing NaOH concentration. As expected, pH 12.5 (0.125 mL of 2.5 M NaOH) was chosen as the best condition to produce a higher number of nanoparticles inside CLPCs. This condition was established to further follow the precipitation of iron oxide particles within CLPCs. After adding NaOH, crystals were kept inside the anaerobic COY from 2 weeks to 6 months.

##### 2.3.1.2 Type 2

In these experiments, iron solution was renewed to avoid its potential depletion due to, i.e., the growth of magnetite in the bulk solution. We followed a similar protocol to that in Type 1. CLPCs (100 crystals of each protein) were placed in the vial containing the master solution and incubated for 4 days (as determined in Type 1 experiments). The precipitation of iron oxides was triggered by increasing the pH to 12.5, adding 0.125 mL of 2.5 M NaOH, and kept inside the anaerobic COY for 10 days (Cycle 1). Then, at least two crystals of each protein were taken for characterization, and the rest was placed in a new vial containing the freshly prepared master solution to start the new cycle (Cycle 2). This process was repeated nine times except for lipase (CLLPCs), because crystals lost their integrity after the second cycle.

#### 2.3.2 Precipitation of Magnetite in the Absence of CLPCs

To evaluate the formation and growth of magnetite nanoparticles in the absence of CLPC (control), a series of experiments were performed following the same protocol as explained above but in the absence of CLPC. Therefore, a vial containing 98,75 mL of master solution was prepared as detailed above, and 1.25 mL of 2.5 M NaOH was added to increase the pH of the master solution to 12.5. After adding NaOH, the sample was homogenized and aliquoted in 10 different bottles. After 14 days (the time equivalent to the full cycle in Type 2 experiments), a sample was collected out of the chamber for evaluation by (HR)TEM.

### 2.4 Characterization

#### 2.4.1 (HR)TEM Sample Characterization

For TEM (FEI-TECNAI T20 at 200 kV and LIBRA 120 PLUS Carl Zeiss, Germany) observation, CLPCs were dehydrated with ethanol and embedded in Epoxy Resin: EMbed-812 from EMS (Electron Microscopy Sciences). Ultrathin sections (50–70 nm) were prepared using a Reichert Ultracut S microtome (Austria, Vienna) after which the cuts were deposited onto G300 Mesh Square Copper (Agar Scientific). The control magnetite nanoparticles were put onto CF200-Cu Carbon Film, Mesh 200 Copper (EMS) grids. The distribution, size, growth, and evolution of formed iron oxides nanoparticles inside the pores of CLPCs and in control samples were analyzed.

The elemental analysis and diffraction patterns (*d*-spacing by using SAED) of the iron oxide nanoparticles were analyzed with high-resolution HR-TEM, FEI TITAN G2 (The Netherlands) and Philips CM20 equipped with energy-dispersive X-ray microanalysis. To determine *d*-spacings from SAED patterns, measurements were taken horizontally and vertically and average to diminish camera distortions.

### 2.5 Statistical Analysis

The size, number, distribution, and diffraction patterns of the nanoparticles were analyzed with ImageJ 1.53e software from TEM micrographs. An average number of 100 nanoparticles per sample were measured to calculate size distribution from at least two different TEM micrographs. For nanoparticle distribution along the CLPCs, TEM micrographs were divided into 100 zones of equal area (182.5 nm^2^). From these 100 zones, we randomly pick three zones near the center of the crystal and another 3 near the border to count and measured all the particles. The statistical analyses were done with OriginPro 2021. The difference between the number of the nanoparticles near the border and in the center was evaluated by the one-way analysis of variance test and the size distribution by the Kruskal–Wallis test. The *p*-values have been compared with the significance level to evaluate the null hypothesis where there were no differences between means or when the null hypothesis indicates that the population means are all equal. A significance level type I error of 0.05 was considered to be the minimum accepted level that denotes a difference between means. The notation that we have included hereafter is * *p* < 0.05, ** *p* < 0.001, and *** *p* < 0.0001 when the differences between means are statistically significant.

Iron oxide nanoparticle distribution inside lysozyme crystals was evaluated from the border to the center of a CLLC along a crystal section. The TEM image was converted to a black and white image and divided the length of the crystal in eight sections of 135 nm (see Figure S3 for details). Then the black/white ratio was calculated using the image analysis software ImageJ 1.53e. The value “0” (black) was indicative of the presence of iron oxide particles, and the value “255” (white) corresponds to the absence of nanoparticles.

## 3 Results and Discussion

**Figure 3:**
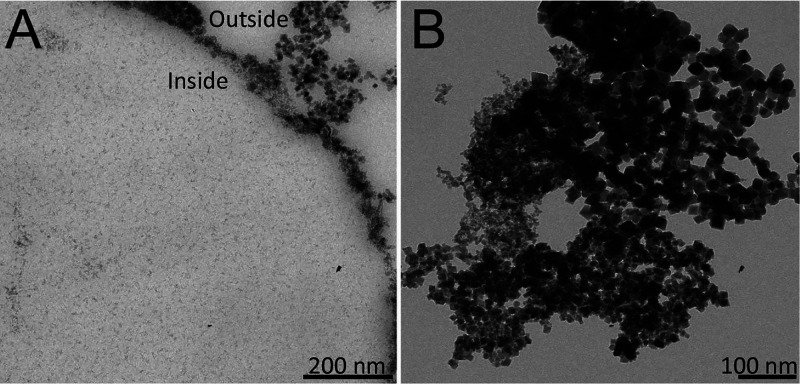
TEM images of (A) iron oxide nanoparticles grown in CLLC experiments (four cycles) inside and outside the protein crystal and (B) in the bulk (protein free) experiment (four cycles).

**Figure 4:**
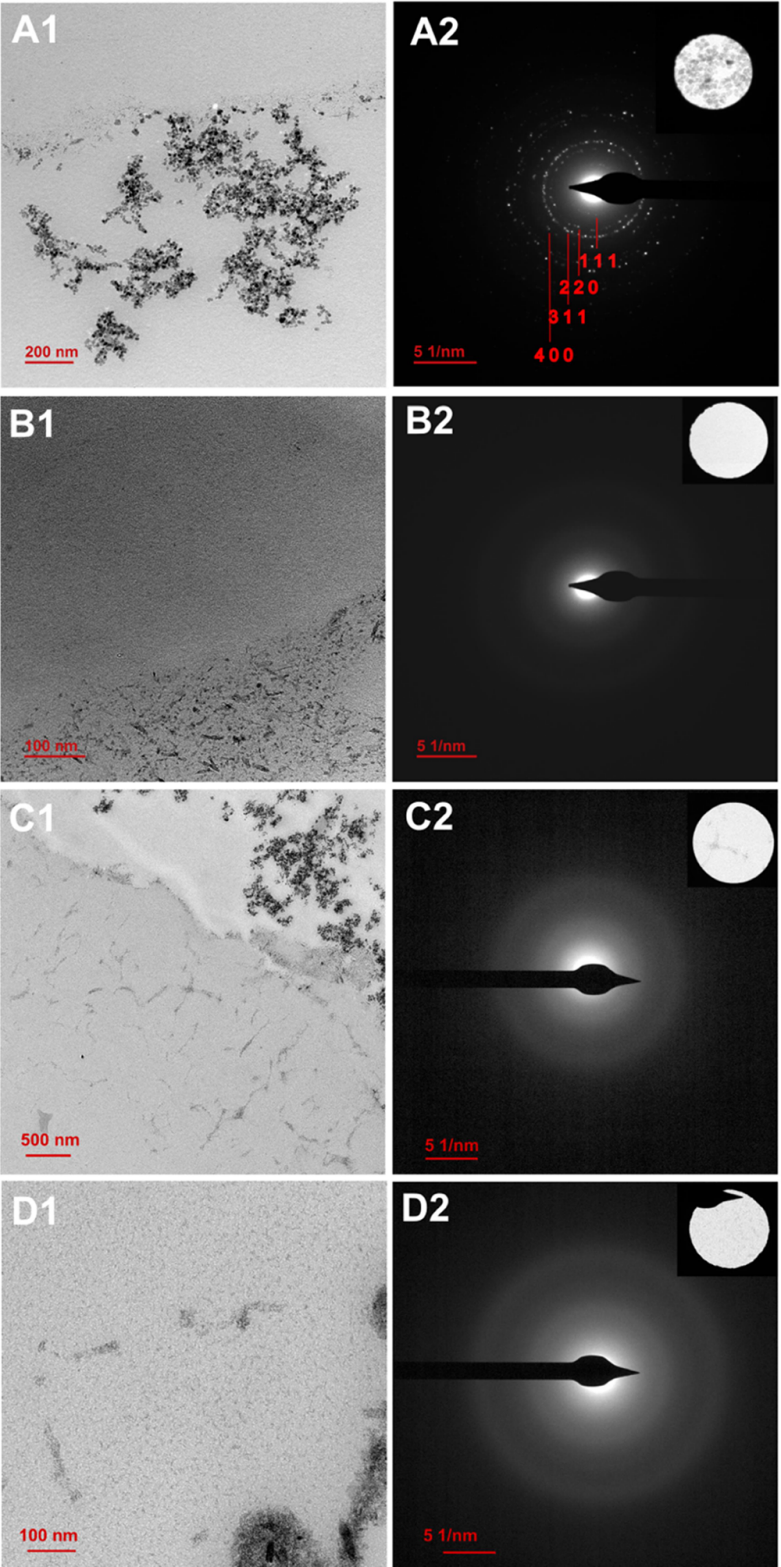
TEM images of magnetite grown outside (A1) and iron oxides nanoparticles inside (B1, C1, and D1) CLLCs after cycle number 2 (A1 and B1), 4 (C1), and 8 (D1). SAED diffraction images are shown in A2, B2, C2, and D2 (insets correspond to the diffraction area). Only crystals obtained outside CLLCs showed SAED diffraction pattern consistent with magnetite (A2) (see also Figure S2).

CLLCs were studied in greater detail as a model to understand the evolution of the confined magnetite precipitation. TEM images of thin slides of CLLCs showed clearly distinct areas (Figure 3A). On one of these areas was the bulk (here referred as outside) in which magnetite crystals of size ∼3–15 nm and similar shape, even a bit smaller than those formed in the control sample (∼3–25 nm) were clearly observed (Figure 3A superior right corner versus Figure 3B). The mineral phase was determined by SAED analyses of those crystals (Figure 4A2), and d-spacing was calculated and compared to that in the relevant literature matching those referenced for magnetite.^28,49^ This precipitation outside CLLCs acted as a positive control, showing that mineral formation was allowed in the system. A second area located along a semicircular border (Figure 3A) showed again the presence of particles of similar size to those obtained in the bulk (outside). This border was delimiting the area of the protein crystal.

Finally, a completely different third area was observed (Figure 3A, inside) in which iron oxide nanoparticles (Fe presence confirmed by EELS) of extremely small size (∼2 nm) were found. It is interesting to note that these nanoparticles were very homogenous in size and were distributed along the whole area inside the CLLCs, and this holds true for all the CLPCs studied. These results show that the conditions (supersaturation) under which this Fe phase was precipitating inside protein crystals were different from those occurring in the bulk. Moreover, given the homogeneity in the size of nanoparticles precipitated inside CLLCs, and considering that the pore size of lysozyme is also of approximately 2 nm,^50^ these results also seem to point out a potential effect of the pore size on the particle formation inside CLLCs.

However, when the same experiment was identically performed but kept for a longer period of time (6 months), no nanoparticles were observed by TEM inside the CLLC. CLLCs did not show any signal of collapse or alteration. However, crystals were still obtained outside of the CLLC, again, showing a size and morphology that compare to that of the crystals formed in the control experiment (protein free) (Figures 3B and S3). These results indicate that those extremely small nanoparticles observed inside the CLLC at shorter period of times were, in fact, Fe-rich metastable intermediates that acted as the iron reservoir. During the time course experiment, and due to the mineral (magnetite) precipitation in the bulk, the supersaturation with respect to the relevant Fe-rich phase decreased outside of CLLCs, forcing the dissolution of these Fe-rich intermediates inside CLLCs.

To further study the process and to avoid Fe depletion in the bulk, a sequential time-lapse strategy was designed in which loading of the crystals with iron solution (4 days) and precipitation in basic media (10 days) were repeated nine times (here referred to as cycles) (Type 2 protocol). CLLPCs and CLGICs were also included in the set of experiments to further study the effect of the pore size on the growth of the Fe-rich phase inside the protein crystals, since they had wider pore channels than that of lysozymes (8.0 nm Ø for CLLPCs and 3.5 nm Ø for CLGICs, as determined from the crystal packing) (Figure 1).

Nanoparticle precipitation in the bulk (outside, Figure 4A1) was observed from the first cycle in all experiments, those nanoparticles having SAED patterns consistent with magnetite (Figure 4A2). On the contrary, nanoparticles inside CLLCs were only observed after the second cycle and held thereafter (Figure 4), and EDX analysis confirmed the presence of iron (Figure S1). These nanoparticles were similar in size (∼2.0 nm) to those obtained following the previous (Type 1) protocol and matched very well the channel diameter of the lysozyme crystals (∼2.0 nm Ø), suggesting a potential physical barrier restraint over nanoparticle growth. SAED patterns only show two diffuse rings that come from the carbon background, suggesting that the 2 nm nanoparticles are amorphous (Figure 4B2,C2,D2).

**Figure 5:**
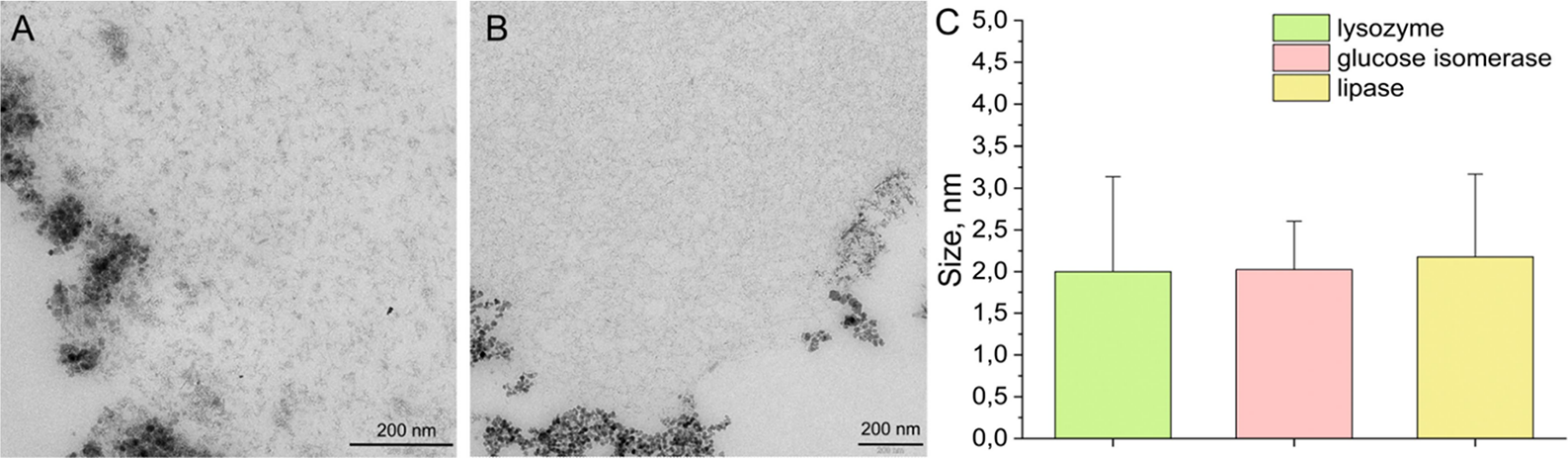
TEM images of iron oxides nanoparticles grown within CLGICs (A) and CLLPCs (B) after two and one cycles, respectively. The plot (C) shows the average nanoparticle particle size for the three proteins after the first cycle.

Similarly, after two cycles of incubation for CLGICs and one cycle for CLLPCs, Fe-rich nanoparticle formation was also detected (Figure 5A,B). TEM images of these nanoparticles showed similar results, in terms of size, homogeneity, and location distribution than those yielded for the nanoparticles inside lysozyme crystals (Figure 5C).

**Figure 6:**
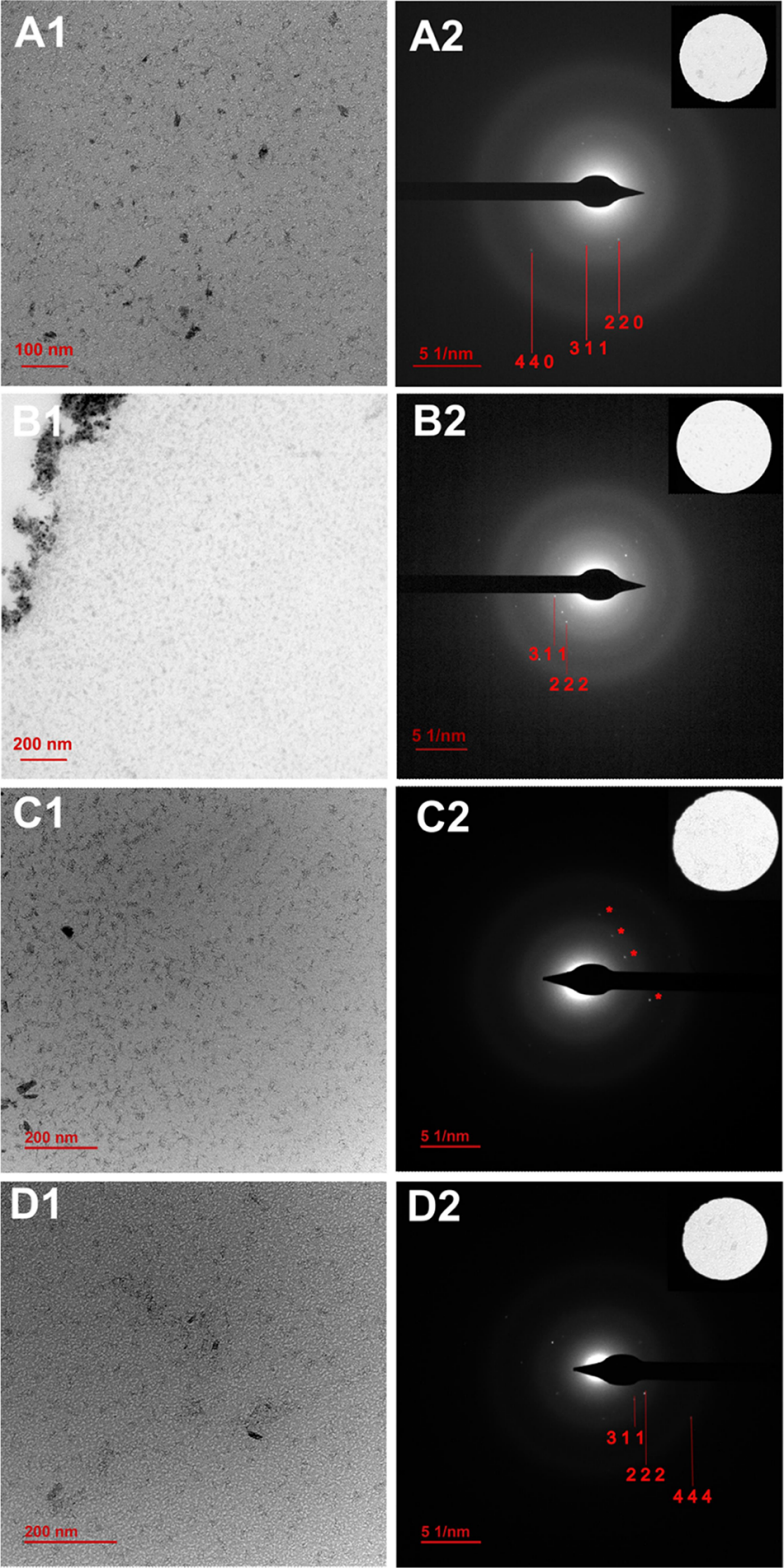
HR-TEM images of magnetite grown inside CLGICs after cycle 2 (A1), cycle 4 (B1), cycle 6 (C1), and cycle 8 (D1) and the corresponding SAED diffraction images of selected regions (insets in A2, B2, C2, and D2). C2 shows single crystal spots corresponding to 111 magnetite reflections. Indexation of all images is shown in Table S1.

**Figure 7:**
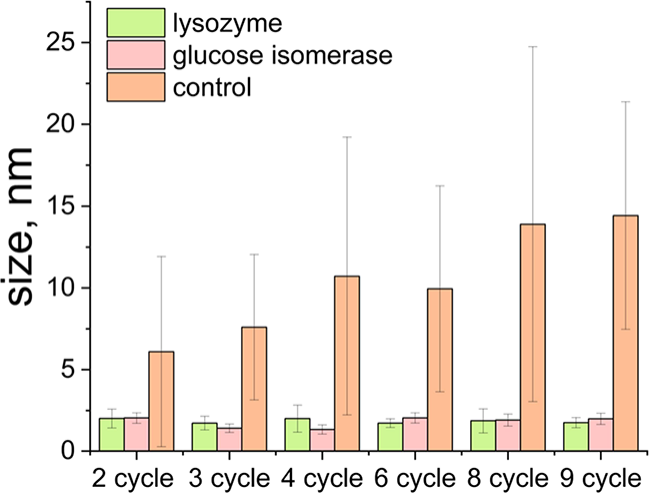
Mean size of magnetite nanoparticles formed inside pores of CLLCs and CLGICs and in solution without CLPCs (control), big standard deviation in the controls appears due to the heterogeneous size of the particles.

SAED results confirmed that the iron oxide nanoparticles formed inside CLGICs were crystals of magnetite from the second cycle (Figure 6 and Table S1). Unfortunately, CLLPCs did not survive more than two cycles of iron oxide precipitation avoiding any further analysis and comparison (Figure S4). In all cases, the size of these magnetite nanoparticles formed inside the protein crystals channels is significantly different from that of the nanoparticles formed outside or in the control experiment, which grew over time (Figure 7). Interestingly, this growth over time that was observed in the magnetite nanoparticles formed outside the protein or in the control experiments was prevented inside the protein crystal channels (Figure 7).

The nanoparticle growth process may be restricted by one and/or the combination of the following circumstances: (1) following upon Fe depletion due to an insufficient Fe flux; (2) by physical barrier constrain caused by the protein crystal porous size; and/or (3) by the stabilization of the nuclei (negatively charged) with the positively charged residues in the protein. It has been previously demonstrated that nuclei can be stabilized because of the electrostatic interaction between the protein positively charged functional groups and the negatively charged mineral surfaces.^51,52^ In fact, while the hydrated surface of magnetite remains basically uncharged at neutral pH as a consequence of the dominant neutral surface species ≡Fe(*II*,*III*)OH, as the pH value increases, Fe(*II*,*III*)OH becomes dominant, and, at even higher pH values, the dominant species are Fe(*II*,*III*)O^–^, being, in these conditions, the surface of magnetite negatively charged^53^ and thus able to interact and be stabilized by positively charged residues in the pore channel.

In the case of lysozyme crystals, once the bulk solution enters the channel system of the protein, a localized Fe-rich phase nucleation is expected following an ionotropic effect triggered by a local high supersaturation with respect to magnetite induced by the acidic residues present in the channel. Those nuclei keep growing as the income of Fe continues, but, once they reach the size of the CLLC pore channel (2 nm), (1) the mineral fills the pore and the surface of the nanoparticles start interacting with the residues in the channel (possibly positive residues) and (2) new Fe incomes are impeded because of pore clogging. The interaction between the mineral and the protein residues in the channel stabilizes the particles and prevents further growth. Under these conditions, Fe-rich particles formed inside lysozyme crystal are not able to grow any further. They stay stable as a metastable phase and may dissolve over time when the bulk becomes undersaturated with respect to magnetite, as it is well known that amorphous phases are more soluble than crystalline ones. Interestingly, our observation of the formation of an Fe-rich metastable phase is consistent with previous results,^15−17^ which observed the formation of a ferrihydrite metastable precursor that later crystallized into magnetite. Therefore, in the case of lysozyme crystals, it seems that the diameter of the channel is determinant for the nanoparticle size and to avoid the transition from the metastable precursor to the crystalline phase.

Different is the scenario for CLLPCs and CLGICs. As in CLLCs, the nucleation of an Fe-rich phase induced by an ionotropic effect is expected due to the presence of acidic residues in the protein channels. However, being the channel pore size larger than in CLLC, two main differences can be pointed out with respect to what occurred in CLLC: (1) the flux of Fe into the protein crystal channel is not blocked, as the size of the nuclei (2–3 nm) is smaller than the diameter of the channel, and (2) the interaction between the mineral surface and the channel protein residues is more limited, as the mineral does not fill the pore. These facts would result in a lack of mineral stabilization and the possibility for a recrystallization of the potential precursor into the more stable phase. SAED-HR-TEM analysis (Figure 6) of the Fe-rich nanoparticles formed inside CLGICs (3.5 nm pore channel) showed that these nanoparticles presented diffraction peaks in all the evaluated cycles. The identification of (111) and (311) crystal faces from the diffraction pattern confirmed the presence of crystalline magnetite. This result is quite interesting since it shows that the nanoparticles are able to restructure from amorphous to crystalline, although a significant size change was not observed. Although, the most usual mechanism for this transformation is a dissolution-precipitation mechanism, in the case of the transformation within CLGICs, the transition from amorphous to magnetite may be triggered by the continuous increase of supersaturation since iron diffusion is not impeded. At this point, we cannot explain why magnetite particles grow to reach the pore size of CLGICs.

**Figure 8:**
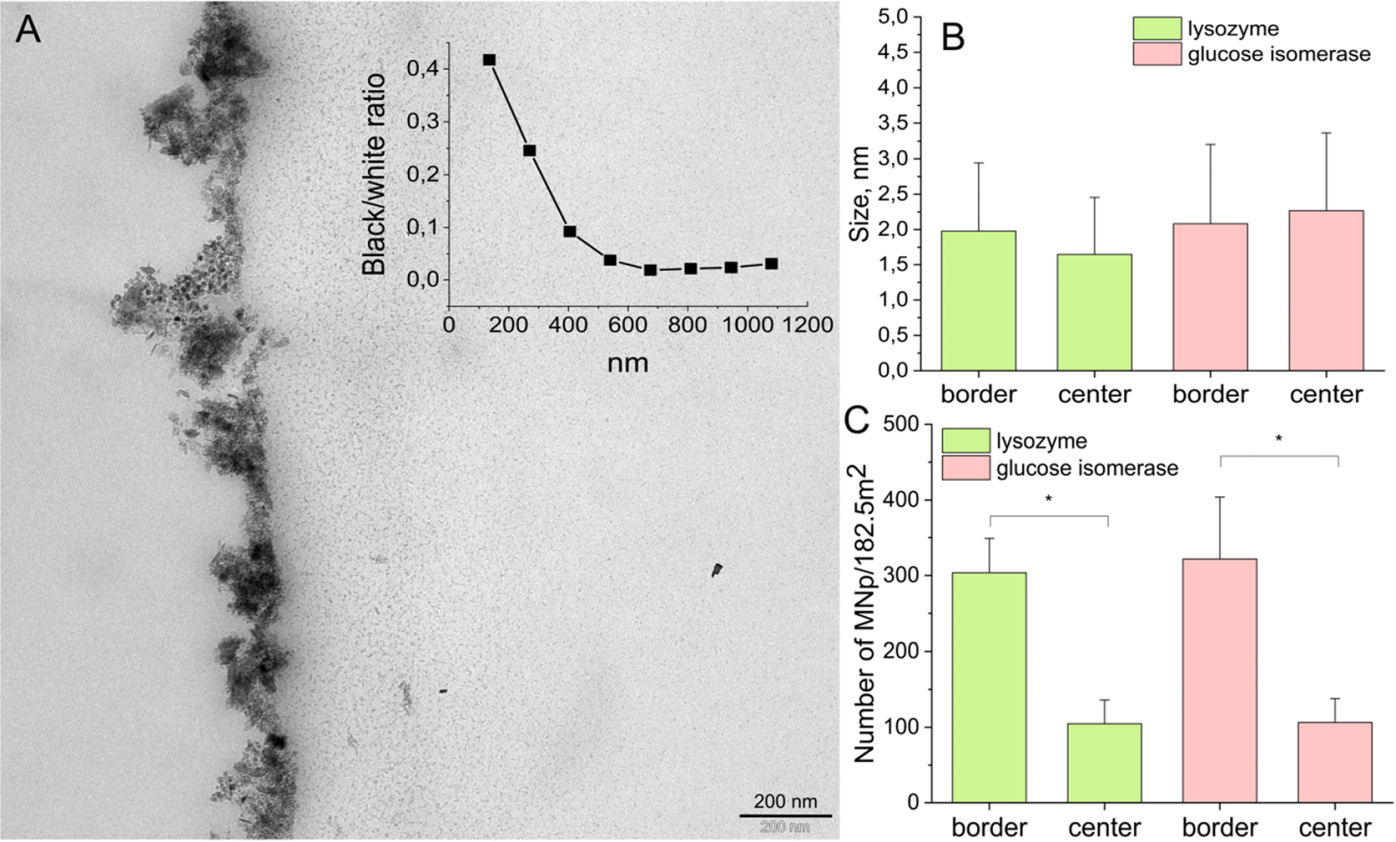
(A) TEM image of CLGICs after eight cycles which illustrates the distribution of magnetite nanoparticles. Particle distribution was calculated along the length of the crystal by determining the black/white ratio in eight regions of 135 nm each and plotted in the insert. (B) Number and (C) size of the magnetite nanoparticles formed near and far from the border of the CLPCs, based on TEM images from the ninth cycle.

The flux of iron from the bulk solution to the protein channels induced a gradient in the concentration of nanoparticles from a higher concentration near the border of the crystal to a lower concentration the farther from the border going inside the protein crystal, as it can be seen in Figure 8A which is a TEM image of CLGICs after 8 cycles. Figure 8B shows that the number of particles near the border is practically the triple with respect to that of the particles inside the protein crystal, that holding true for both CLLCs and CLGICs. No statistically significant differences were observed in the size of the nanoparticles from each group among the two crystalline proteins, but differences were observed between the size of the crystals inside and in the border, pointing again to an effect of confinement during the precipitation of iron oxides particles (Figure 8C).

## 4 Conclusions

Herein, we have studied the aqueous coprecipitation of iron oxide salts at room temperature inside CLPCs. The use of CLPCs has allowed us to study how chemical and physical factors can influence magnetite mineralization in a confined space. Our results have shown that nucleation of an Fe-rich phase occurs inside the protein channels in all cases, but the channel diameter size is important to stabilize metastable precursor, thus preventing their recrystallization into the more stable phases, magnetite. Smallest pore sizes impose physical barriers that make more likely the (1) stabilization of the metastable precursors by the chemical interaction of the nanoparticles with the protein functional groups and (2) restrained Fe diffusion into the channel system. A bigger pore size allows the transition from metastable precursor to crystalline particles as observed with CLGICs. These results have shown the drastic effects that crystallization inside CLPCs can have in the mineralization of magnetite. Finally, we have also proven that CLPCs can be interesting biomimetic porous materials to study crystallization processes in confined spaces.

## Supporting Information Available

Figure S1: Elemental analysis of CLLCs, Figure S2 *d*-spacing values of the diffraction pattern shown in Figure 4A2; Table S1. *d*-spacing values of the diffraction pattern shown in Figure 6A2,B2,C2,D2; Figure S3: Protocol for iron oxide nanoparticle distribution determination; Figure S4: magnetite crystals grown in the bulk (protein free); Figure S5: HR-TEM and SAED of CLLPCs (PDF)
