# Supplementary material for: Magnetite Mineralization inside Cross-Linked Protein Crystals
Source: Cryst Growth Des. 2023 Apr 28;23(6):4032–40. doi: 10.1021/acs.cgd.2c01436 (PMC10251750; doi:10.1021/acs.cgd.2c01436)
Supplement: Supplementary file 1 — cg2c01436_si_001.pdf [file cg2c01436_si_001.pdf]

Magnetite mineralization inside cross-linked protein crystals.

Mariia Savchenko,[1,2, 4] *Victor Sebastian,*[3,] *Modesto Torcuato Lopez-Lopez,*[4,5]*, Alejandro Rodriguez-Navarro,*[6]*Luis Alvarez de Cienfuegos,*[*1,5] *Concepcion Jimenez-Lopez,*[*7] and *José Antonio Gavira*[*2]

[1]Universidad de Granada,Departamento de Química Orgánica, Facultad de Ciencias,Unidad de Excelencia de Química Aplicada a Biomedicina y Medioambiente (UEQ), 18002 Granada, Spain.

[2]Laboratorio de Estudios Cristalográficos, Instituto Andaluz de Ciencias de la Tierra (Consejo Superior de Investigaciones Científicas-Universidad de Granada), Avenida de las Palmeras 4, 18100 Armilla, Granada, Spain. UEQ.

[3]Department of Chemical Engineering and Environmental Technology; Instituto de Nanociencia y Materiales de Aragón (INMA), CSIC-Universidad de Zaragoza, Zaragoza 50009, Spain; Networking Research Center on Bioengineering Biomaterials and Nanomedicine(CIBER- BBN), Madrid 28029, Spain.

[4]Universidad de Granada, Departamento de Física Aplicada, Facultad de Ciencias, Spain.

[5]Instituto de Investigación Biosanitaria ibs.GRANADA.

[6]Universidad de Granada, Departamento de Mineralogía y Petrología. Facultad de Ciencias, Spain.

[7]Universidad de Granada, Departamento de Microbiología. Facultad de Ciencias, Spain.

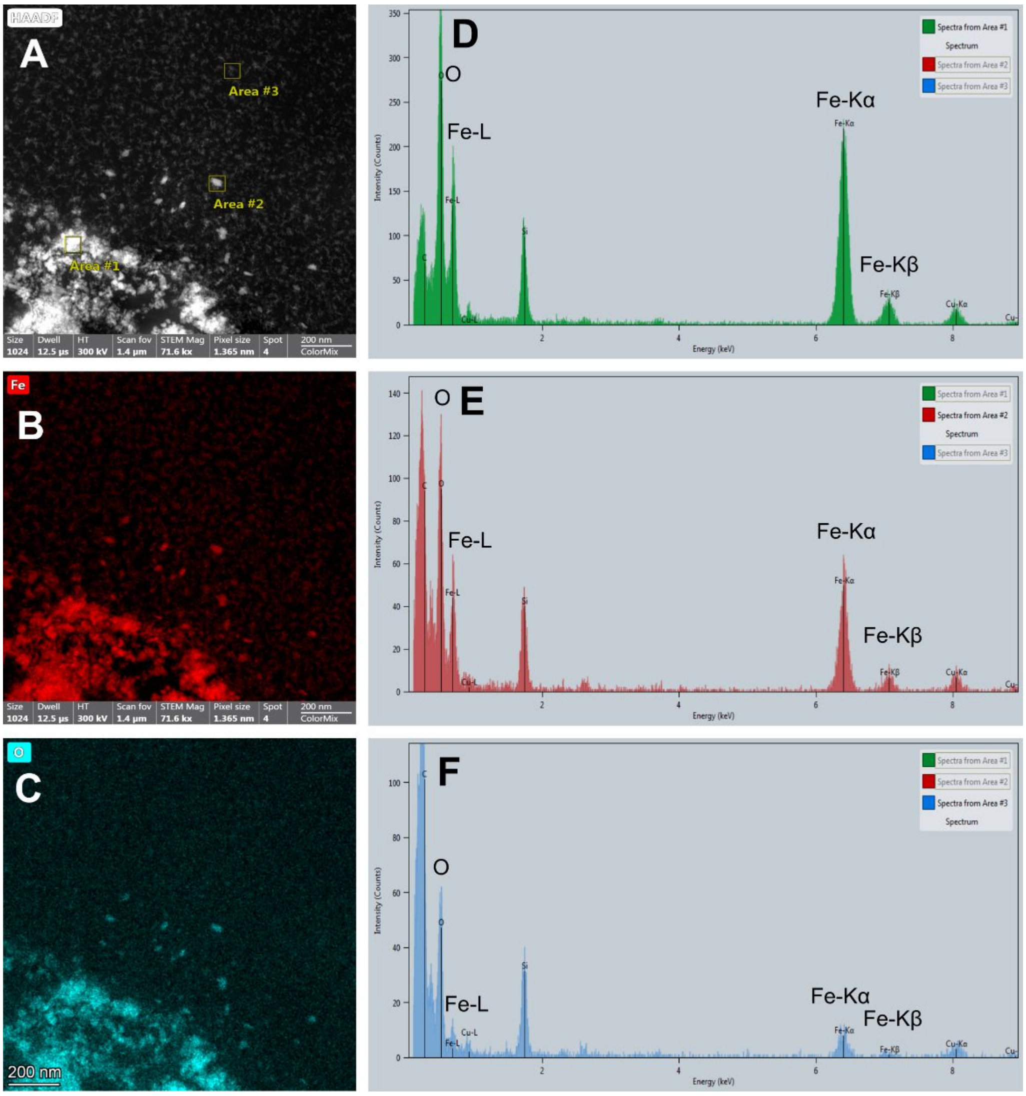

Figure S1. Elemental analysis of CLLCs from cycle number 6 showing the presence of iron in the three regions, inside the crystal (A), near the border (B) and at the border (C).

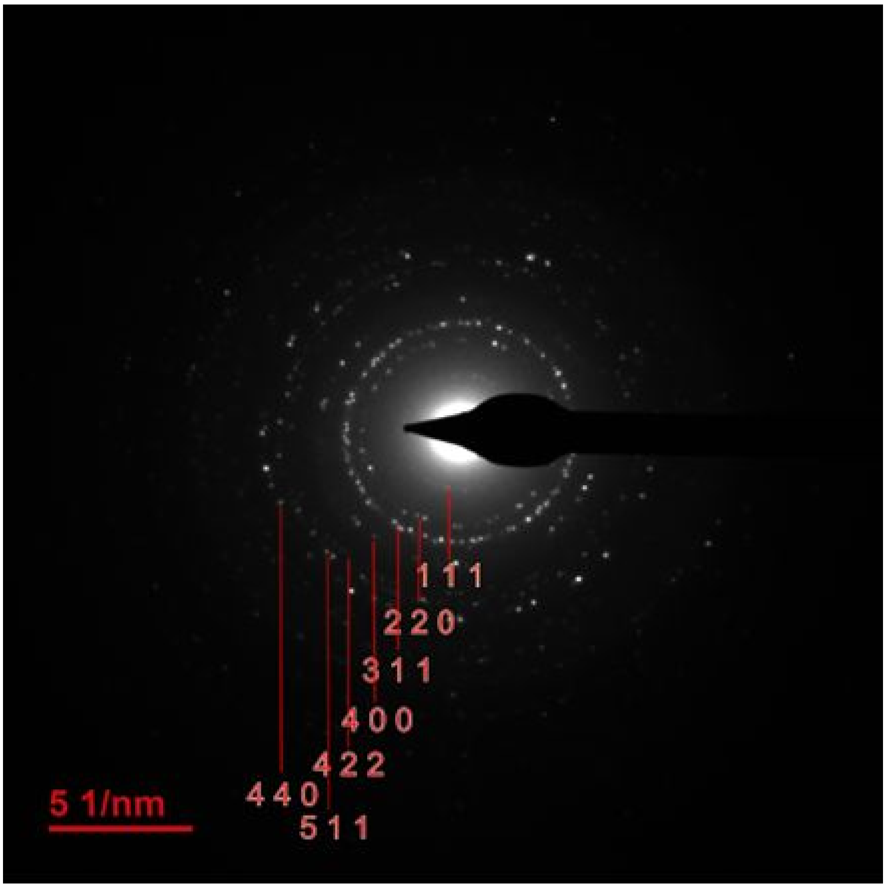

lenght	Å	d-spacing*	H	K	L
4.16	4.81	4.85	1	1	1
6.68	2.99	2.97	2	2	0
7.90	2.53	2.53	3	1	1
			2	2	2
9.63	2.08	2.1	4	0	0
11.56	1.73	1.72	4	2	2
12.32	1.62	1.62	3	3	3
12.32	1.62	1.62	5	1	1
13.03	1.53	1.49	4	4	0

* Downs et al. (1993)

Figure S2. d-spacing values of the diffraction pattern shown in Figure 4 A2 of magnetite obtained outside CLLCs.

A2

lenght	Å	D-SPACING *	H	K	L
7,17	2,79	2,97	2	2	0
8,04	2,49	2,53	3	1	1
13,38	1,49	1,49	4	4	0

B2

8,04	2,49	2,53	3	1	1
8,25	2,43	2,43	2	2	2

C2

	4,81	4,85	1	1	1

D2

7,91	2,53	2,53	3	1	1
8,40	2,38	2,43	2	2	2
17,04	1,17	1,21	4	4	4

Table S1. d-spacing values of the diffraction pattern shown in Figure 6 A2, B2, C2, D2.

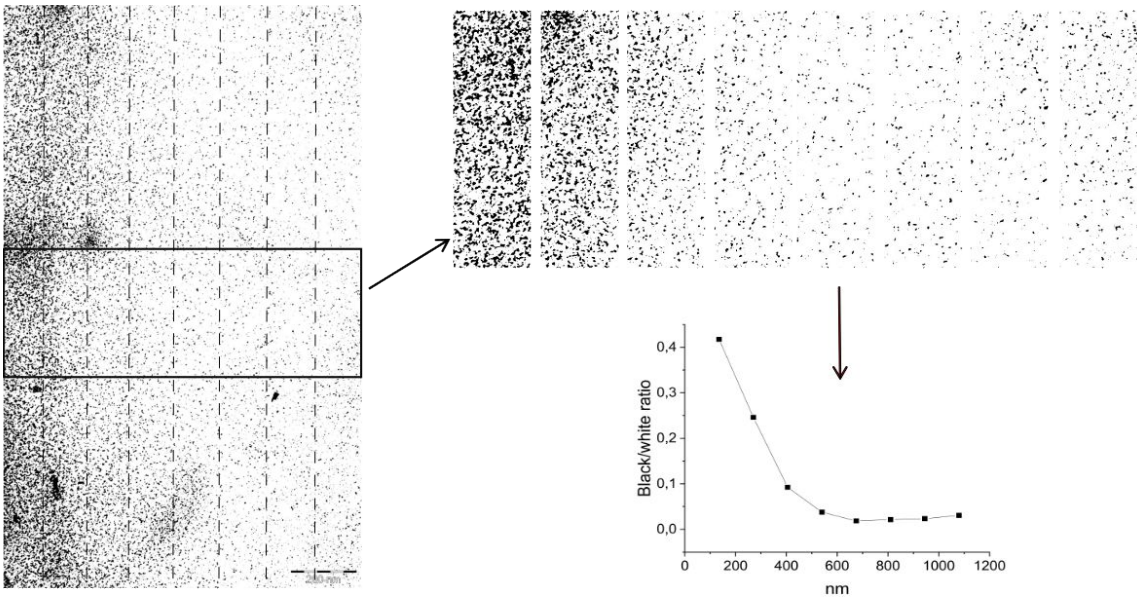

Figure S3. Summary oftheprotocol to determine the distribution of the nanoparticles from the border to the center of the crystal.

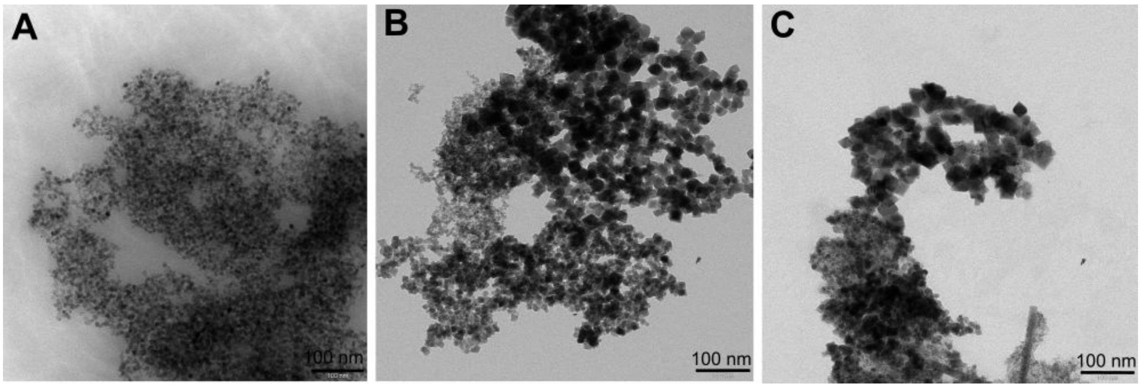

Figure S4 TEM images of magnetite particles grown in the bulk (protein free) and used as reference.

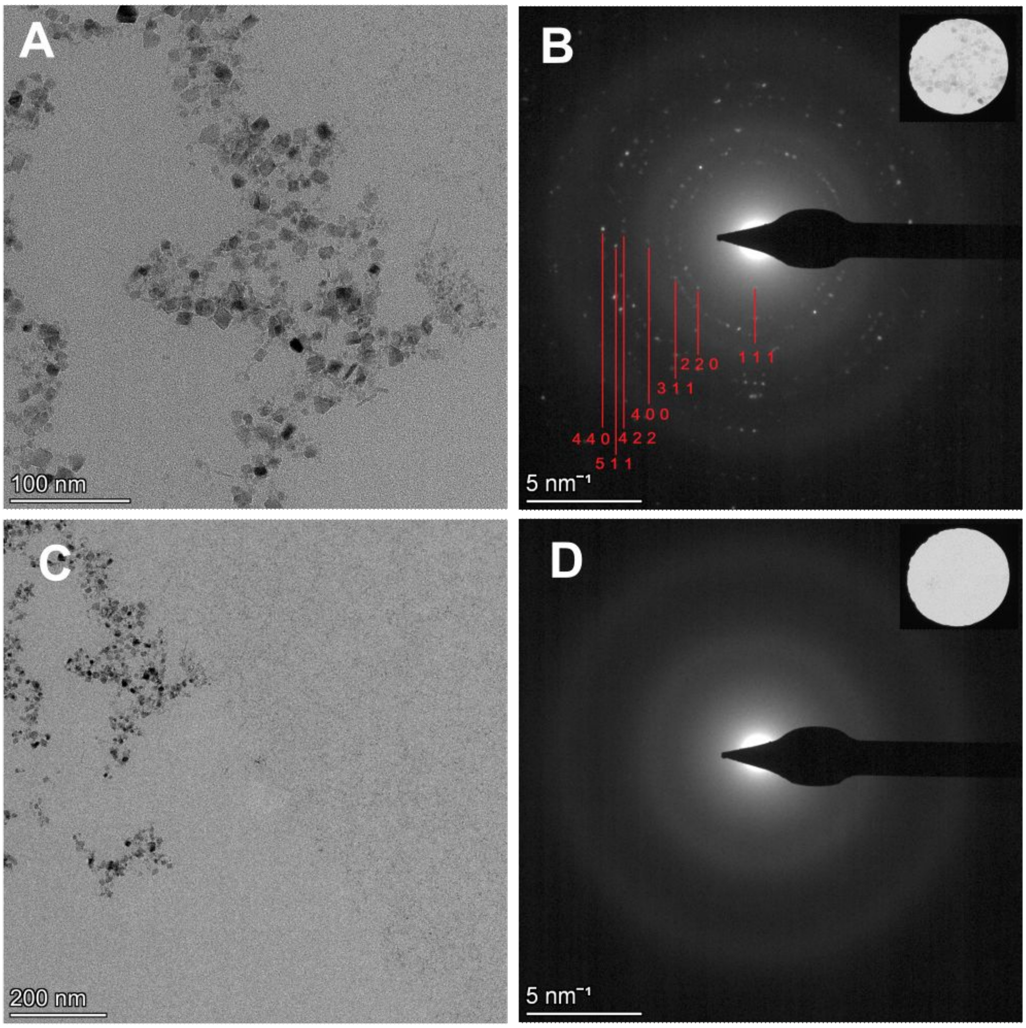

Figure S5. TEM images of magnetite obtained after one cycle outside (A) and iron oxides nanoparticles inside (C) CLLPCs. B and D correspond to the SAED diffraction images of selected regions (insets).
